# Self-propelled droplet transport on shaped-liquid surfaces

**DOI:** 10.1038/s41598-020-70988-x

**Published:** 2020-09-11

**Authors:** Gaby Launay, Muhammad Subkhi Sadullah, Glen McHale, Rodrigo Ledesma-Aguilar, Halim Kusumaatmaja, Gary G. Wells

**Affiliations:** 1 Smart Materials and Surfaces Laboratory, Faculty of Engineering and Environment, Northumbria University, Newcastle upon Tyne, NE1 8ST UK; 2 Department of Physics, Durham University, Durham, DH1 3LE UK

**Keywords:** Mechanical engineering, Chemical physics, Fluid dynamics

## Abstract

The transport of small amounts of liquids on solid surfaces is fundamental for microfluidics applications. Technologies allowing control of droplets of liquid on flat surfaces generally involve the generation of a wettability contrast. This approach is however limited by the resistance to motion caused by the direct contact between the droplet and the solid. We show here that this resistance can be drastically reduced by preventing direct contact with the help of dual-length scale micro-structures and the concept of “liquid-surfaces”. These new surfaces allow the gentle transport of droplets along defined paths and with fine control of their speed. Moreover, their high adhesion permits the capture of impacting droplets, opening new possibilities in applications such as fog harvesting and heat transfer.

## Introduction

The transport of small quantities of liquid on a solid surface is inhibited by the resistance to motion caused by the contact between the liquid and the solid. To overcome such resistance, motion can be externally driven through gradients in electric fields^1,2^, temperature^3–5^, light^6,7^ and pressure^8^, or structural topography combined with vibration or phase change^9,10^, but these all inconveniently involve the input of external energy. Alternatively, gradients in physical shape and wettability—the conical shape of cactus spines^11^ and the wettability of butterfly wings^12^—occur naturally and can be engineered into surfaces to create self-propelled motion^7,13–18^. However, such self-propelled motion to date has limited success in overcoming the inherent static resistance to motion of the liquid contact with the solid. This resistance can be significantly reduced by introducing an intermediary lubricant layer^19,20^ whose shape can be modulated by an underlying topography to achieve better control over the interaction of these surfaces with target liquids^21–25^. Here we demonstrate the self-propulsion of droplets on a shaped-liquid surface, where a lubricant layer is combined with a heterogeneous topography. The resultant surface contains an inherent gradient in liquid-on-liquid wettability with minimal resistance to motion and long range directional self-propelled droplet transport. Moreover, the liquid-liquid contact enables impacting droplets to be captured and transported, even when the substrate is inverted. These design principles are highly beneficial for droplet transport in microfluidics, self-cleaning surfaces, fog harvesting and in heat transfer.

## Results and discussion

An ideal design of a system to move small quantities of fluid should not require complex propulsion mechanisms based on continual input of energy. It should neither present large threshold sticking forces to be overcome nor limit the distance of transport. The ability to provide fine control of speed of motion, to adhere and transport liquids without boundary walls and to do so in multiple orientations, whether uphill or inverted, would provide additional benefits. Motion without energy input, has led to a focus on surfaces with a gradient in physical properties and in the use of topographic features^18^. These features provide a gradient in the wetting properties of the solid surface, whilst retaining a uniform surface chemistry, and create a self-propulsion force on droplets. However, direct contact between the droplet and the solid is needed to drive motion, and this introduces static and dynamic friction forces. The consequent minimum self-propulsion forces required for transport necessitate large gradients in the topographic texture. Large gradients, in turn, lead to limited control of a droplet’s velocity when transported over long distances. To overcome these limitations, we hypothesized that contact with the solid could be completely replaced by another liquid, but in a manner that still allows a gradient in wettability to exist. To do so, we used a dual length scale substrate providing both a liquid surface and a gradient in its surface texture. This enables liquid-on-liquid wetting to drive the motion of droplets, where the driving mechanism is mediated by an asymmetry built into an underlying solid substrate which shapes the liquid surface. To illustrate liquid-on-liquid wetting, first consider placing a water droplet on a thin film of silicone oil that coats a flat hydrophobic solid surface. Because silicone oil completely wets water, the oil will cloak the droplet and isolate it from the solid surface^20,26^.

**Figure 1:**
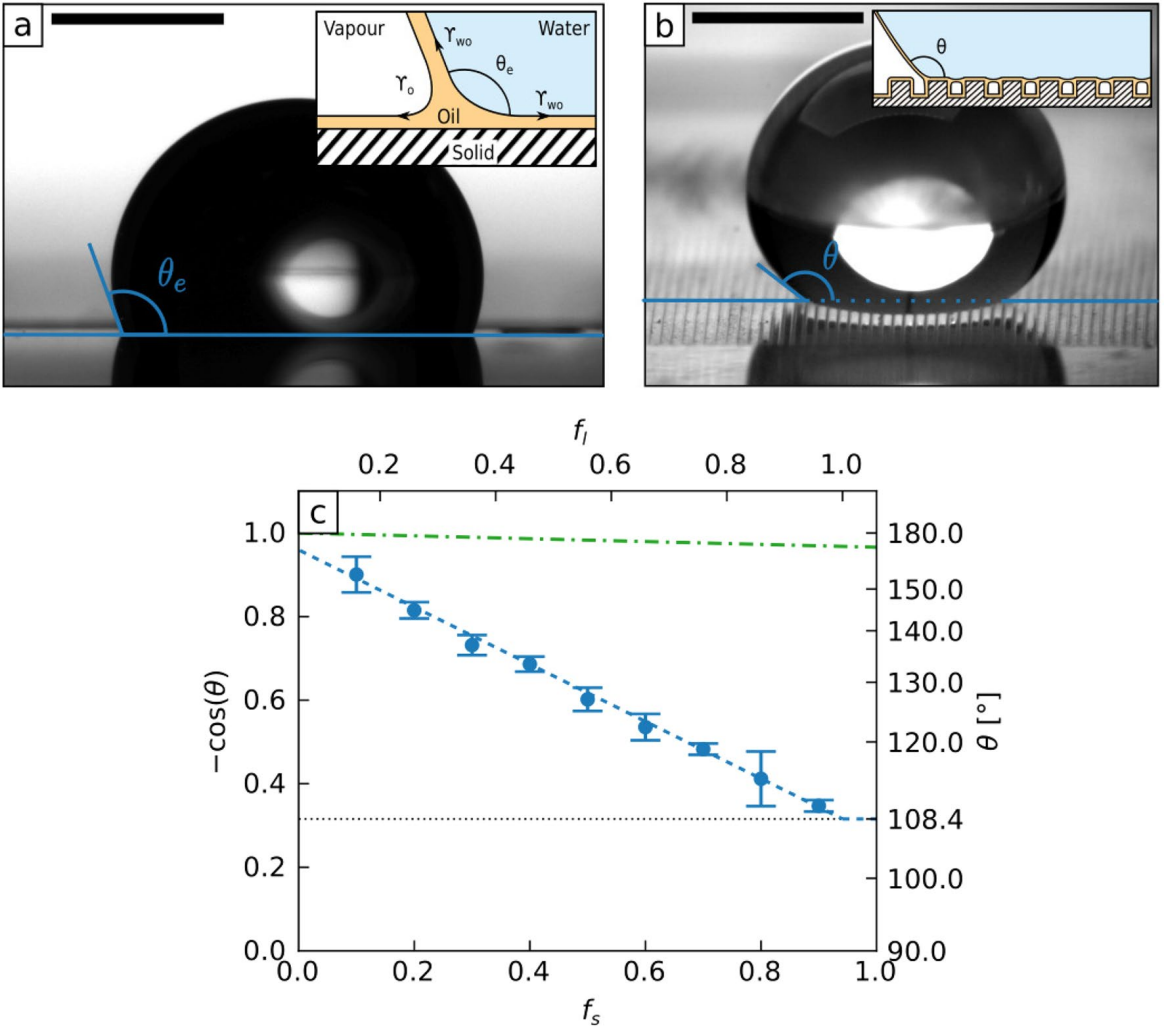
Wetting on liquid surfaces. (**a**) Water droplet on a low-hysteresis liquid surface obtained by imbibing an hydrophobic nano-particles coating with silicon oil. The scale bar is 1 mm. (inset) Diagram presenting the surface tensions acting at the droplet edges. (**b**) Water droplet on a liquid surface shaped with rectangular rails. The fraction of the sample covered by the rails is $f_s=0.18$. Pockets of air are trapped underneath the droplet, modifying its apparent contact angle. The scale bar is 1 mm. (inset) Illustration of the expected oil distribution. (**c**) Droplet apparent contact angle, $\theta$, as a function of the rail fraction $f_s$, for $5\, \upmu {\hbox {L}}$ water droplets deposited on shaped liquid surfaces. Error bars are the standard deviation over eight measures. (Blue) dashed line is Eq. (2) with $f_l = f_s + 0.059$. (Green) dashed-dotted line is the Cassie–Baxter equation for a textured oil-free super-hydrophobic surface. Additional measurements are shown on Supplementary Fig. S4.

Even though there is no direct contact of the droplet with the solid surface, it still exhibits an apparent contact angle, $\theta _e$, due to the interfacial tensions pulling on the outer and inner surfaces of the oil layer at the droplet’s edge (see inset to Fig. 1a). This leads to an effective Young’s Law for liquid-on-liquid wetting^27,28^

1 $$\begin{aligned} \cos \theta _e = \frac{\gamma _{oa} - \gamma _{wo}}{\gamma _{eff}} \end{aligned}$$

where $\gamma _{eff} = \gamma _{wo} + \gamma _{oa}$. Here, $\gamma _{oa} = {19.8}\ {\hbox {mNm}}^{-1}$ and $\gamma _{wo} = 38\ {\hbox {mNm}}^{-1}$ are the surface tensions of the oil-air and water-oil interfaces and predict $\theta _e = 108.4^{\circ }$. If the oil is replaced by another immiscible liquid having a negative spreading power on water, the liquid may still isolate the droplet from the solid surface but does not encase the droplet and so $\gamma _{eff} = \gamma _{wa}$. Since Eq. (1) has no explicit dependence on the solid surface, it can be regarded as a definition of wettability for an immiscible liquid surface in the thin-film limit. To create liquid-on-liquid wetting with a surface capable of being shaped, we introduce a small length scale solid texture using a hydrophobic nanoparticle-based coating. This allows the silicone oil to be retained and provides a continuous liquid surface^19,29^. The apparent contact angle of a droplet of water on this liquid surface is $\theta _e = 109.3 \pm 0.7^{\circ }$ consistent with Eq. (1) (Fig. 1a) and confirms the absence of direct contact with the solid surface. Measurements show that the droplet on the liquid surface exhibits a very low contact angle hysteresis ($\Delta \theta \approx 1^{\circ }$) and sliding angle ($\theta _s <1^{\circ }$ for a $5 \, \upmu {\hbox {L}}$ droplet, see Supplementary Information for measurement protocol) thus removing the principal constraint on self-propulsion using a gradient surface. This approach allows us to create a conformal liquid coating on a solid substrate which can be textured at a larger length scale and so unlocks the ability to create wetting contrasts and gradient wettability with a liquid surface. We now consider the static wetting of a water droplet on liquid surfaces with textures designed to control wettability. For the larger length scale, we used arrays of rectangular cross-section rails ${60}\, \upmu {\hbox {m}}$ high and ${75}\, \upmu {\hbox {m}}$ apart, and with solid surface area fractions $f_s$ between 0.1 and 0.9. We applied the nanoparticle-based coating as our second, smaller, length scale to enable the silicone oil to create a liquid surface that conforms to the large-scale solid texture. We observed, using a camera and a macro lens, that a water droplet rests on top of the rails leaving pockets of air underneath^30^ (Fig. 1b and inset), with low contact angle hysteresis ($\Delta \theta \approx {1}^{\circ }$) and a contact angle (viewed across the rails) which increases with decreasing rail fraction (Fig. 1c). This is reminiscent of the Cassie–Baxter state for superhydrophobic surfaces^31,32^, but here the contact angle, $\theta$, is determined by the liquid [Eq. (1)]^33^

2 $$\begin{aligned} \cos \theta = f_l \cos \theta _e - (1 - f_l) \end{aligned}$$

and $f_l \approx f_s$ is the liquid surface area fraction. This prediction agrees with measurements and provides an excellent fit by including a small correction $f_l=f_s+0.059$ (Fig. 1c). This correction is consistent with the estimated nanoparticle-based coating thickness in our experiments ($\approx \, {2}\, \upmu {\hbox {m}}$, giving a correction of $\approx \, 0.053$). This suggests the droplet is in a mixed wetting state suspended above a composite liquid and air surface. To further confirm there is no contact with the solid we considered the same substrate without silicone oil. Crucially, because of the high contact angle of ${165}^{\circ }$ on this oil-free surface compared to the lower apparent contact angle of ${108.4}^{\circ }$ on those with oil, the variation of contact angle with rail fraction is significantly weaker (compare dashed and dashed-dotted lines in Fig. 1c). This implies that the gradient in wettability for droplet self-propulsion is significantly stronger for the liquid surface and amplifies the effect of the liquid surface in removing pinning (see Supplementary Information). To verify the droplet recognizes contrasts in wettability on a composite liquid–air surface, we placed a droplet at the boundary between two regions of different rail fraction. The droplet spontaneously moved to the region of higher wettability defined by Eq. (2) (Supplementary Fig. S5 and movie S1).

**Figure 2:**
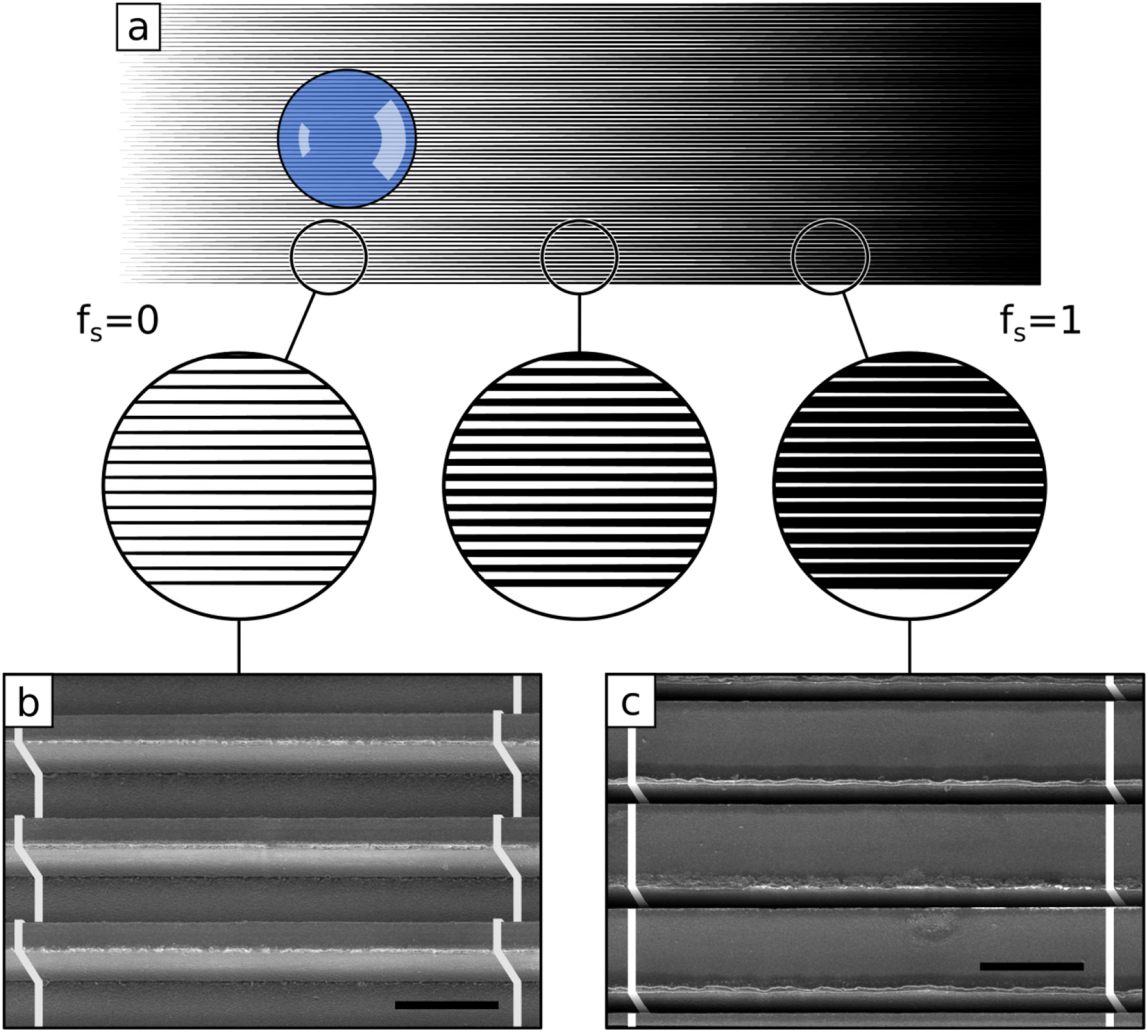
Rail geometry used to create gradients of wettability on super-hydrophobic and liquid surfaces. (**a**) Parallel rails with divergent width along their length. The center-lines of two consecutive rails are separated by ${75}\, \upmu {\hbox {m}}$. The solid surface area evolves from $f_s=0$ on the left to $f_s=1$ on the right. (**b**,**c**) SEM images of the divergent rails at two different positions. The scale bars are ${75}\, \upmu {\hbox {m}}$.

We now consider the self-propulsion of a water droplet on a liquid surface induced by a designed gradient in wettability. For the larger length scale, we fabricated an array of 2 cm long and ${60}\, \upmu {\hbox {m}}$ high rails, where the width of each rail increases along its length. At the low surface fraction end of the substrate, the tops of adjacent rails are ${75}\, \upmu {\hbox {m}}$ apart (Fig. 2).

The liquid surface area fractions $f_l$ increases linearly from 0 to 1 at a rate $\alpha = {0.05}\,{\hbox {mm}}^{-1}$ along the direction of the rails (Fig. 2a). Without the liquid surface provided by silicone oil, a droplet remains pinned to the solid despite the wettability gradient (Supplementary Fig. S6).

**Figure 3:**
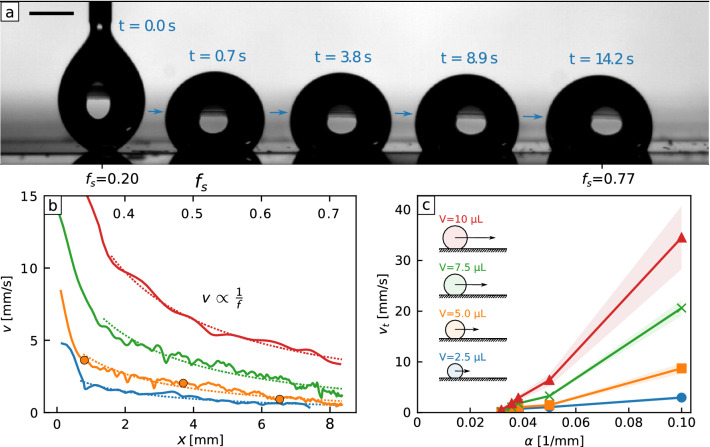
Self-propulsion on a textured gradient liquid surface. (**a**) ${5}\, \upmu {\hbox {L}}$ water droplet propelled on a textured liquid surface. The gradient of rail fraction ($\alpha = {\mathrm {d}} f_s / {\mathrm {d}} x = {0.05}\,{\hbox {mm}}^{-1}$) induces the wettability gradient driving the droplet. The scale bar is 1 mm. (**b**) Droplet speed during motion for different volumes: ${2.5}\, \upmu {\hbox {L}}$ (blue), ${5}\, \upmu {\hbox {L}}$ (yellow), ${7.5}\, \upmu {\hbox {L}}$ (green) and ${10}\, \upmu {\hbox {L}}$ (red). (Yellow) circles correspond to the droplet at three of the times presented in (**a**) ($t=0.7$ s, $t=3.8$ s and $t=8.9$ s). After an initial transient regime due to the deposition of the droplet, the velocity decreases in 1/*f* along the sample (dashed lines as fits). (**c**) Typical speed $v_t$ of water droplets as a function of gradient of rail fraction $\alpha$ and droplet volumes (blue circles: ${2.5}\, \upmu {\hbox {L}}$, orange squares: ${5}\, \upmu {\hbox {L}}$, green crosses: ${7.5}\, \upmu {\hbox {L}}$ and red triangles: ${10}\, \upmu {\hbox {L}}$).

On the shaped liquid surface, however, we observed a sustained self-propulsion over the pattern (Fig. 3a).

It is well-known that droplets on liquid-infused surfaces tend to displace the lubricant, which eventually change the properties of the surface (higher pinning and contact angle hysteresis). In the present case, the available oil is reduced to a minimum, as most of it is secured in the nano-porosity. This allows to keep the surface properties constant along numerous repetitions. For example, we have been able to propel 20 successive droplets on the sample presented Fig. 3a without seeing any change in the droplet velocities. This propulsion can also be designed into complex pathways, as illustrated by transport along a curved path (Supplementary Fig. S9 and Movie S3). Returning to our design criteria and the desire for controlled long distance transport and fine control of droplet speed, we discovered an inverse linear relationship between speed of motion and rail fraction $v \propto 1/f_s$ (Fig. 3b). The slowing-down dynamics occurs for a range of droplet volumes ($V = 2.5 {-} 10\, \upmu {\hbox {L}}$) and wettability gradients ($\alpha = 0.03 {-} 0.1\, {{\mathrm{mm}}}^{-1}$). By balancing the driving force $F_d \propto \alpha \gamma _{oa} R^2$ due to the wettability gradient along the droplet’s perimeter (where *R* is the droplet’s base) with viscous resistance $F_v \propto \mu _o f_s R v$ from the edge of the drop, we find $v \propto \gamma _{oa} \alpha R/\mu _o f_s$ (Supplementary Information). This explains the observed slowing-down with increasing rail fraction (Fig. 3b) and predicts an increase of speed with wettability gradient and droplet volume, which we confirmed by measuring the typical droplet speed, $v_t$, defined as the speed at the surface point where $f_s = 0.5$ (Fig. 3c). Our measurements show self-propulsion ceases below a rail fraction gradient $\alpha \approx {0.03}\,{\hbox {mm}}^{-1}$ implying the presence of a small pinning force (Fig. 3c).

**Figure 4:**
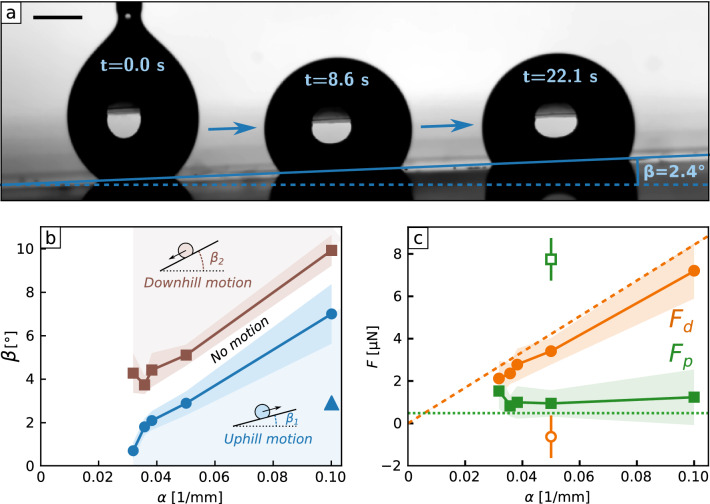
Strength of gradient induced self-propulsion. (**a**) ${5}\, \upmu {\hbox {L}}$ water droplet propelled uphill on a textured liquid surface tilted at an angle $\beta = 2.4^{\circ }$. The scale bar is 1 mm. (**b**) Critical sliding angles $\beta$ as a function of rail fraction gradient $\alpha$, for ${5}\, \upmu {\hbox {L}}$ water droplets. (blue) circles stand for the critical tilting angles $\beta _1$ at which droplets stop moving uphill and (brown) squares stand for the critical tilting angles $\beta _2$ at which droplets stop moving downhill. Dim areas represent the standard deviation calculated on 12 measurements. Between those two critical angles, the driving force is too small to overcome the pinning force and the droplet remains motionless. The (blue) triangle highlights the configuration depicted in (**a**). (**c**) Driving force $F_d$ and pinning force $F_p$ calculated from the critical angle measurements presented in (**b**). (orange) circles are the driving force for liquid (filled symbols) and super-hydrophobic (open symbol) surfaces. (green) squares are the pinning force for liquid (filled symbols) and super-hydrophobic (open symbol) surfaces. Dim areas represent the standard deviation propagated from the measurement of the critical angles. Dashed (orange) and dotted (green) lines are the theoretical predictions of respectively the driving force and the pinning force (see text).

To measure this force, we tested the ability of droplets to move up inclined surfaces (Fig. 4a). It is to be noted that we do not observe oil drainage upon tilting, which can be explained by the fact that all the easily removable oil has been washed away by the water-jet during the surface preparation. We observed upwards self-propulsion below an inclination angle $\beta _1$, implying that the driving force outweighs gravity and pinning (bottom region in Fig. 4b), but above an angle $\beta _2$ droplets run downhill (upper region in Fig. 4b). From the conditions for mechanical equilibrium, the driving force is $F_d = \frac{1}{2} \rho _d V g ( \sin \beta _2 + \sin \beta _1)$ and the pinning force is $F_p = \frac{1}{2} \rho _d V g ( \sin \beta _2 - \sin \beta _1)$. We deduce $F_p \approx {1.11 \pm 0.25}\upmu {\hbox {N}}$ across the range of rail fraction gradients, similar to the small value estimated from contact angle hysteresis measurements, $F_p \approx {0.48}\upmu {\hbox {N}}$ (Supplementary Information). Fig. 4c shows the measured driving force also agrees well with the simple theoretical model (Supplementary Information). Overall, these measurements confirm that pinning is overcome by the driving force over a broad range of wettability gradients and droplet volumes. With such small gradients (down to $\alpha \approx {0.03}\,{\mathrm{mm}}^{-1}$) sustained transport is possible over long distances (here $L = 1/\alpha \approx {30}\,{\mathrm{mm}}$). Furthermore, because of the small pinning force, droplets can also be moved at low speeds (down to ${0.067}\,{\mathrm{mm\,s}}^{-1}$ for a ${5}\, \upmu {\hbox {L}}$ droplet). In contrast, without the shaped liquid surface (Fig. 4c), we find that the pinning force is considerably larger and cannot be overcome by the driving force. In addition to reducing pinning and allowing self-propulsion, the liquid surface significantly increases the ability to capture impacting droplets. The number of bounces of a droplet before it adheres to a solid upon impact depends on the kinetic and surface energy remaining after energy dissipation because of spreading and retraction^34,35^.

**Figure 5:**
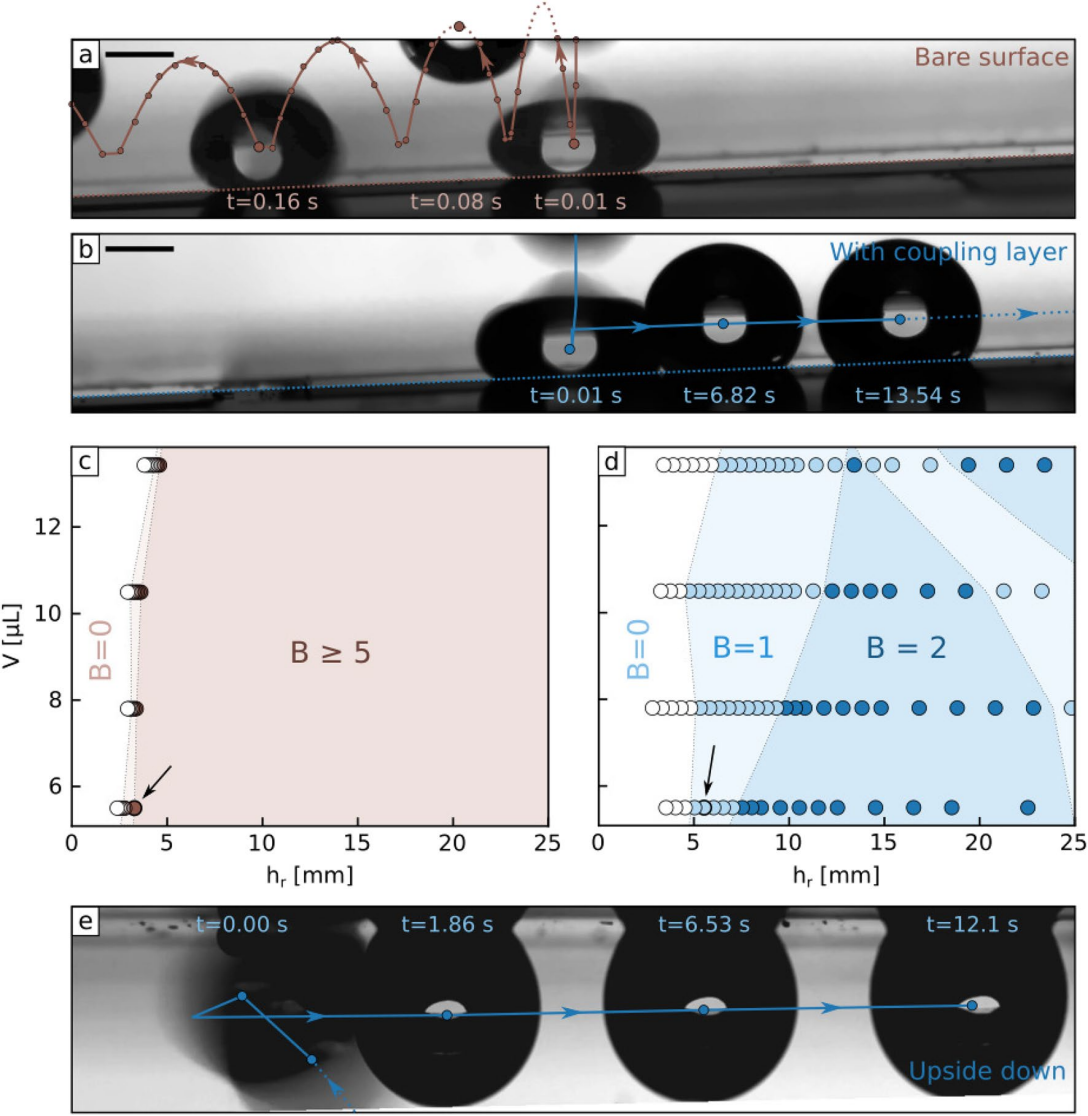
Impact, capture and transport of droplets. (**a**) ${5}\, \upmu {\hbox {L}}$ water droplet impacting on a tilted ($\beta = 2.33^{\circ }$) super-hydrophobic textured surface. Circles are the successive measured positions of the droplet. Due to the low normal adhesion, the droplet bounces and is transported downhill by the effect of gravity. (**b**) Same as (**a**) but for a liquid surface. The higher energy dissipation during impact and the strong wettability gradient allow the droplet to be captured rapidly and transported uphill. (**c**) Number of bounce *B* as a function of droplet volume and release height on a super-hydrophobic tilted surface. The black arrow highlights the configuration depicted in (**a**). (**d**) Similar as (**c**) but for a liquid surface. For all points on this plot, the droplets are successfully captured and moved uphill. The black arrow highlights the configuration depicted in (**d**). (**e**) Hanging droplet, captured and transported on an inverted shaped liquid surface.

On a superhydrophobic surface, the small contact area and low dynamic friction of a droplet leads to a bouncing rate that rapidly increases with increasing droplet impact speed (which we varied by setting the initial release height $h_r$) (Fig. 5a,c), making droplet capture and transport difficult. In contrast, our shaped liquid surfaces increase dissipation upon impact through viscous friction^36^ and have stronger adhesion due to the wettability of the liquid surface. As such, they prevent bouncing over a broad range of release heights and promote capture and subsequent transport via self-propulsion, even uphill (Fig. 5b,d). The droplet dynamics at the moment of impact is also rich: the wettability gradient breaks the symmetry of the impact^36^ and leads to an asymmetrical droplet deformation. The specific mechanisms at play however remain to be unraveled. Figure 5e shows that these surfaces are also capable of capturing and transporting hanging droplets, impacting the surface from below (see also Supplementary Figs. S7 and S8).

## Conclusion

The shaping of liquid surfaces, enabled by a dual length-scale structure, is an advantageous approach to creating wettability contrasts for droplets on liquid surfaces. In this work we used these principles to design liquid surfaces with strong gradients in wettability and low resistance to droplet motion. On a shaped liquid surface, it is possible to gently transport droplets with ease over long distances along defined paths and with fine control of their speed. The design principles presented here provide a foundation for new types of microfluidic devices and, because of the ability to capture and transport impacting droplets in any orientation, may also have wider applications, such as fog harvesting and heat transfer.

## Methods and materials

### Surface fabrication process

Micro-structured surface are manufactured by photolithography. Glass wafers are cleaned prior to the process, using IPA and acetone rinses. To promote the photoresist adhesion, wafers are etched using a reactive-ion etcher (RIE) and bounded with HDMS (Microchem). Surfaces are then spin-coated 30 s at 1750 rpm with a photoresist (SU8-2025 from Microchem), to obtain a layer of ${60}\,{\upmu }{\hbox {m}}$. After a pre-10 min at ${95}\,^{\circ }{\mathrm{C}}$), patterns are transposed from a chrome mask (JD Photo Data) on the surface using a mask aligner (at an UV power of ${300}\,{\hbox {mJ/cm}}^2$). The extra photoresist is removed using EC solvent (Microchem) after a post-exposure bake (6 min at ${95}\,^{\circ }{\mathrm{C}}$). Surfaces are then subjected to a last bake (10 min at ${170}\,^{\circ }{\mathrm{C}}$) to finalize the cross-linking process. The structures obtained are 60 $\upmu {\mathrm{m}}$ high and can be as small as 10 $\upmu {\mathrm{m}}$ wide.

The hydrophobic porous layer is deposited by spraying a commercial solution (Glaco Mirror Coat Zero) of silanized nano-particles. SEM images show that this process creates a conformal porous layer on the micro-structures (Supplementary Fig. S1). At this point, the surface is a super-hydrophobic patterned surface, on which water droplets lie in Cassie–Baxter state (Supplementary Fig. S2). 20 cSt silicone oil is imbibed into the surface by dip-coating it at ${0.1}\,{\hbox {mms}}^{-1}$. This result in an oil layer thickness between 2.6 and 60 $\upmu {\mathrm{m}}$^37^ that fills the micro-structures and prevents the formation of air pockets underneath the droplet (Supplementary Fig. S2). To obtain a liquid surface, we remove the oil from the grooves using a water jet ($\approx {10}\,{\hbox {ms}}^{-1}$ for at 20 s). At this point, only a small amount of oil is kept in place in the porosity of the nano-particle layer. The success of this operation is ensured by verifying that deposited droplets are in Cassie–Baxter state (Supplementary Fig. S2) and exhibit a very low contact angle hysteresis.

### Contact angle measurements

Static contact angles are measured using side visualizations of droplets and an in-house drop shape analyzer software (PyDSA). The robustness of the measurements is ensured by performing repeats and by comparing different methods of contact angle measurements.

The contact angle hysteresis is measured using the inflation–deflation method. A droplet of 4 $\upmu {\hbox {L}}$ is first deposited on the surface and put in contact with a small syringe needle. The side-view of the droplet is recorded using a camera while the droplet is inflated and then deflated slowly through the needle. The evolution of the base radius and contact angles are extracted from the resulting video using PyDSA. From those measurements, the advancing and receding contact angles are deduced as the contact angles for which the base radius begins to respectively increase and decrease. The contact angle hysteresis is then calculated as the difference between the advancing and receding angles. For a liquid surface, measurements indicate that the CAH remains low ($\Delta \theta = {0.66 \pm 0.57}^{\circ }$ on 20 measurements) and is independent of the solid fraction in the range $f_s \in [0.1, 0.9]$.

## Supplementary information

Supplementary Movie S1

Supplementary Movie S2

Supplementary Movie S3

Supplementary Movie S4

Supplementary Information
