# Supplementary material for: Self-propelled droplet transport on shaped-liquid surfaces
Source: Sci Rep. 2020 Sep 11;10:14987. doi: 10.1038/s41598-020-70988-x (PMC7486897; doi:10.1038/s41598-020-70988-x)
Supplement: Supplementary file 5 — Supplementary Information [file 41598_2020_70988_MOESM5_ESM.pdf]

# Self-Propelled Droplet Transport on Shaped-Liquid Surfaces

## Supplementary information

Gaby Launay[1], Muhammad Subkhi Sadullah[2], Glen McHale[1], Rodrigo Ledesma-Aguilar[1], Halim Kusumaatmaja[2], Gary G. Wells[1,*]

[1]Smart Materials & Surfaces Laboratory, Faculty of Engineering & Environment, Northumbria University, Newcastle upon Tyne, NE1 8ST, UK
[2]Department of Physics, Durham University, Durham, DH1 3LE, UK
*email: gary.wells@northumbria.ac.uk

May 23, 2020

## 1 Surface fabrication

**We present here visualizations of the conformal hydrophobic layer and of the effect of the quantity of imbibing oil on the state of sessile droplets.**

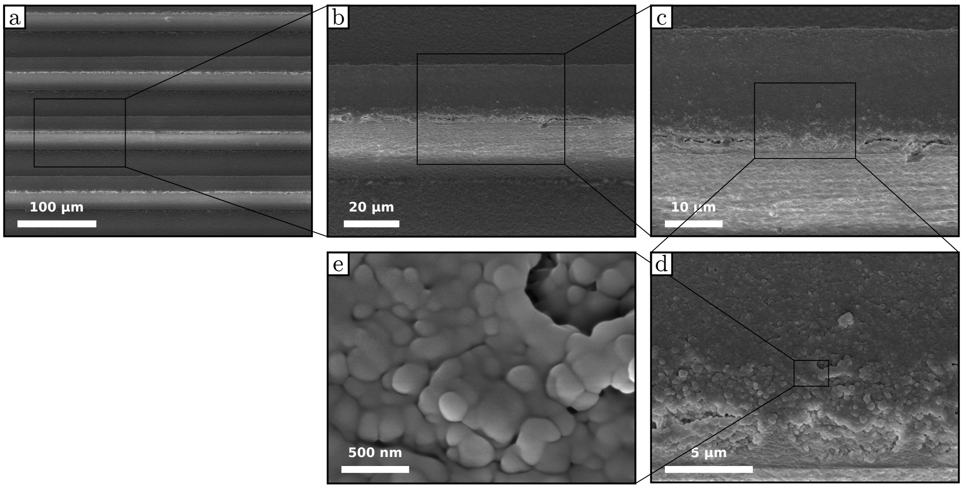


Figure S1: SEM images of the micro-structures manufactured by photolithography and coated with a conformal layer of silanized nano-particles. (a) Parallel rails obtained by photolithography. (b)-(d) Successive close-ups. (e) Close-up of the top of a rail highlighting the nano-particles of the porous layer.

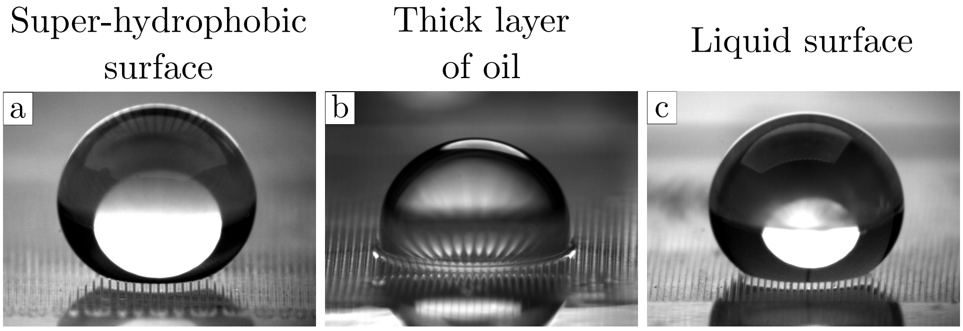


Figure S2: 5 µl droplets on patterned surfaces. (a) Super-hydrophobic patterned surface. The droplet lies in Cassie-Baxter state on the top of rails. (b) After dip-coating the surface in silicone oil, the oil completely fills the micro-structures. The contact angle hysteresis is very low and the droplet highly mobile, but the amount of oil prevents the formation of pockets of air underneath the droplet. (c) After removing the oil from between the rails, we obtain a shaped liquid surface that exhibits a high droplet mobility, while still allowing the formation of air pockets underneath the droplet.

## 2 Sliding angle measurement

**Protocol for sliding angle measurement on liquid surfaces** Figure S3 presents the protocol used to measure the sliding angle of water droplets on liquid surfaces. In this study, the surface tilting angle is increased by steps

of 0.1°, giving a sliding angle with an accuracy of ±0.05°.

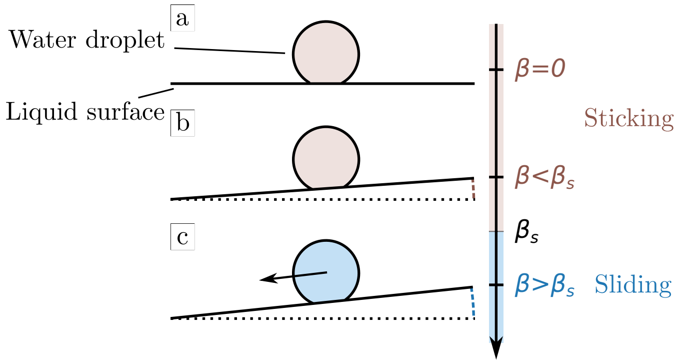


Figure S3: Schematic of the sliding angle measurement protocol: (a) a 5 µL water droplet is placed on a horizontal ($\beta = 0$) liquid-surface with a constant solid fraction (no wettability gradients). (b) The surface is slowly titled by steps of 0.1°. For small values of $\beta$, the droplet remains in place due to the contact angle hysteresis. (c) When the tilting angle exceeds the sliding angle $\beta_s$, the droplet begins to slide along the slope. The tilting angles before and after droplet sliding allow to get a measure of the sliding angle with an accuracy of ±0.05°.

# 3 Apparent contact angle evolution with the solid fraction

**We present here additional measurements of the droplet apparent contact angle as a function of the solid fraction $f_s$.**
Fig. 1c of the main manuscript shows that the apparent contact angle depends linearly of the solid fraction $f_s$ for a droplet on rails with uniform fraction. Fig. S4 indicates that this is also the case on rails with divergent width, for both pinned and moving droplets. For high solid fractions ($f_s > 0.7$), the pocket of air expected to be sustained underneath the droplet are sometimes filled with oil. This explains the decrease of the apparent contact angle to $\theta_e$ (predicted by equation (1) of the main manuscript) for some measurements in this region.

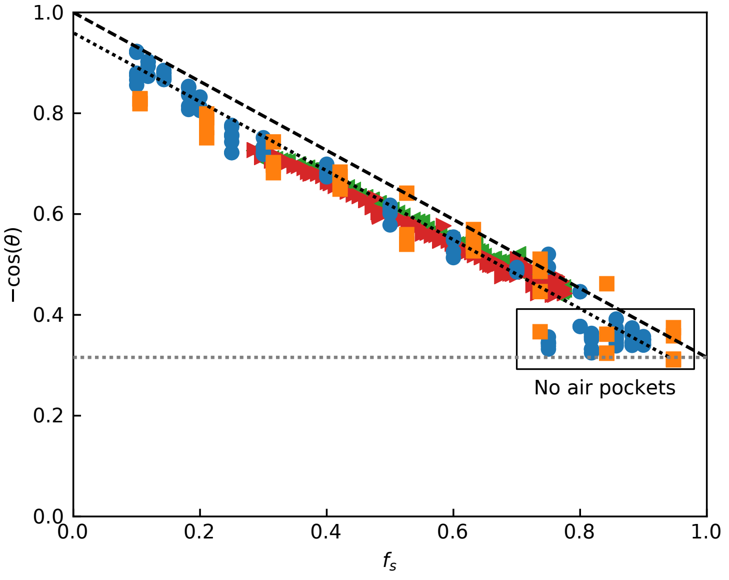


Figure S4: Droplet apparent contact angle as a function of the solid fraction $f_s$. (blue) circles: static droplets deposited on rails with an uniform solid fraction. (orange) squares: droplets pinned in place at different positions on rails with divergent width. (red) right-pointing triangles and (green) left-pointing triangles: respectively left and right contact angle of a droplet moving on rails with divergent width. Dashed line: equation (2) of the main manuscript, using the solid fraction $f_s$ and $\theta_e = 108.4°$. Dotted line: equation (2) of the main manuscript, using the corrected liquid fraction $f_l = f_s + 0.059$. The black box highlights the configurations where air pockets underneath the droplet are filled with oil.

# 4 Droplet response to a wettability contrast on a shaped liquid surface

**A droplet deposited between two regions of different rails fraction spontaneously moves towards the region of higher wettability (higher fraction).**

See also Movie S1.

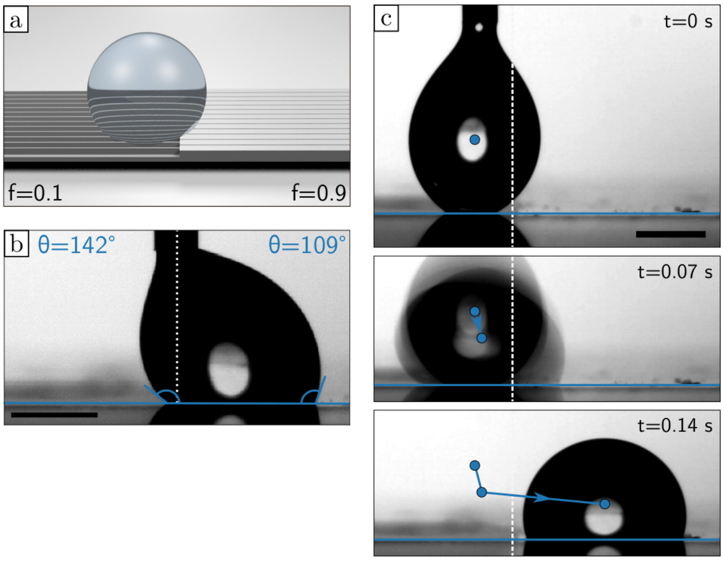


Figure S5: Droplet rapid motion due to a discontinuity in wettability of a shaped liquid surface. (a) Illustration of a droplet on a discontinuity of solid fraction. The solid fraction is $f_s = 0.1$ on the left and $f_s = 0.9$ on the right. (b) Droplet held in place on the discontinuity to highlight the difference in wettability between the two sides. (c) Droplet released on the discontinuity and moving rapidly towards the region of higher wettability (higher solid fraction). The scale bars are 1 mm.

## 5 Droplet on patterned super-hydrophobic surfaces

**We compare here the effect of the wettability gradient on a droplet motion for a super-hydrophobic and a liquid surface.**
See also Movie S2.

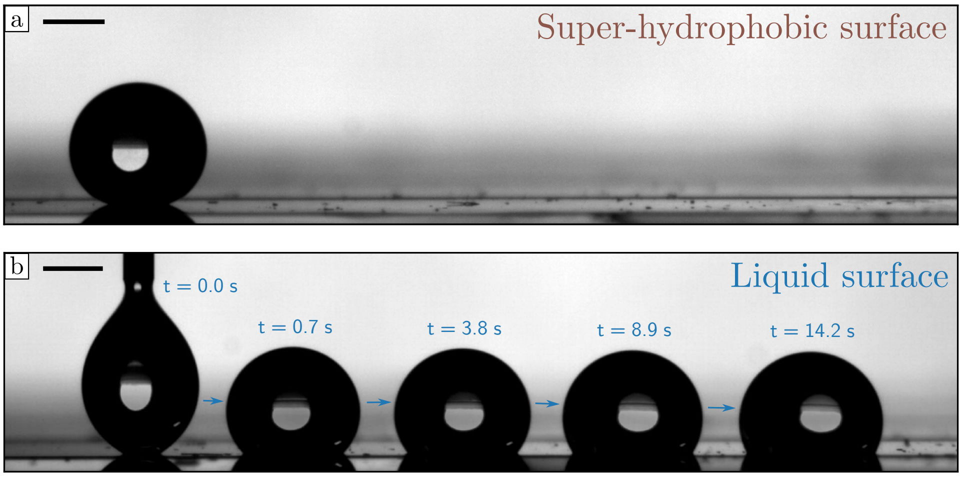


Figure S6: Droplets deposited on surfaces with a gradient of solid fraction ($\alpha = 0.05\,\mathrm{mm}^{-1}$). (a) Super-hydrophobic surface. The gradient of wettability is not strong enough to overcome the pinning force and the droplet remains motionless. (b) Liquid surface. The increased gradient of wettability and decreased pinning force allow the droplet to move towards the high wettability regions (*i.e.* lower apparent contact angle as defined by equation (1) of the main manuscript). The scale bars are 1 mm.

# 6 Force balance model

**We derive here the model used to estimate the driving and pinning forces for a droplet on a shaped liquid-surface.**

## 6.1 Driving force

The elementary force per unit length acting at a specific point of the contact line of a droplet (and towards the droplet center) reads:

$$g = (\gamma_{oa} + \gamma_{wo}) \cos\theta \tag{S1}$$

The resulting force acting on the droplet can be obtained by integrating this elementary force on the contact line. In the case of a droplet on a continuous, linear gradient of solid fraction $\alpha = \frac{\partial f_s}{\partial x}$, and assuming the droplet to have a rectangle footprint of length $R$ and width $kR$, the capillary force acting on the droplet is

$$F_d = 2kR\,[g(x=-R) - g(x=R)] \tag{S2}$$

Using equation (S1) and equation (2) from the main manuscript, equation (S2) simplifies in

$$F_d = 8k\gamma_{oa}\alpha R^2 \tag{S3}$$

## 6.2 Viscous resistance

As soon as a droplet is put in movement, a force due to viscous friction opposes its motion. Different mechanisms participate to this viscous dissipation: the dissipation (i) in the droplet bulk, (ii) in the oil layer underneath the droplet and (iii) at the droplet contact line. The dissipation in the droplet bulk is expected to generate a resisting force

$$F_{v,b} = \int_{S_b} \mu_w \frac{\partial v_b}{\partial y}\, dS_b \propto f_s \mu_w v R \tag{S4}$$

with $S_b$ the surface of contact between the droplet and the surface and $v_b$ the velocity in the droplet bulk. Assuming the velocity at the top of the oil layer to be $v_o(y=h) = vh\mu_w/R\mu_o$ (with $h$ the oil thickness), the force due to the dissipation in the layer of oil is

$$F_{v,o} = \int_{S_b} \mu_o \frac{\partial v_o}{\partial y}\, dS_b = \int_{S_b} \frac{h}{R}\mu_w \frac{\partial v}{\partial y}\, dS_b \propto f_s \mu_w v R \tag{S5}$$

Finally, using a Taylor expansion of the Cox-Voinov equation, we can show that the force due to the dissipation at the contact line reads

$$F_{v,c} \propto f_s \mu_o v \ln\left(\frac{R}{L_o}\right) R \tag{S6}$$

with $L_o$ the slip length.

Those three forces scale as $f_s v R$. However, the force linked to the dissipation at the contact line is expected to dominate in our configuration thanks to the viscosity ratio ($\mu_o/\mu_w = 20$) and the value of $\ln(R/L_o) \approx 10$ (for $R = 1\,\text{mm}$ and $L_o = 10\,\text{nm}$). Being the dominant dissipation force, $F_{v,c}$ will be called $F_v$ in the sequel.

### 6.3 Pinning force

Because of chemical and topological heterogeneities, a droplet on a surface always exhibits a contact angle hysteresis. In the context of moving droplets, this hysteresis leads to a pinning force that need to be overcome. Still assuming a droplet with a rectangular footprint, the pinning force can be deduced from the advancing and receding contact angles (respectively $\theta_a$ and $\theta_r$), as

$$F_p = kR\left(\gamma_{wo} + \gamma_{oa}\right)\left(\cos\theta_a - \cos\theta_r\right) \tag{S7}$$

The difference in contact angle cosine can be rewritten as:

$$\cos\theta_a - \cos\theta_r = -\,2\sin\left(\frac{\theta_a + \theta_r}{2}\right)\sin\left(\frac{\theta_a - \theta_r}{2}\right) \tag{S8}$$

$$= -\,2\sin\theta_{eq}\sin\left(\frac{\Delta\theta}{2}\right) \tag{S9}$$

with $\Delta\theta = \theta_a - \theta_r$ the contact angle hysteresis and $\theta_{eq}$ the equilibrium contact angle, estimated to be equal to $(\theta_a + \theta_b)/2$. Introducing equation (S9) into equation (S7) leads to:

$$F_p = -kR\left(\gamma_{wo} + \gamma_{oa}\right) 2\sin\theta_{eq}\sin\left(\frac{\Delta\theta}{2}\right) \tag{S10}$$

### 6.4 Terminal velocity

A droplet on a wettability gradient reaches its terminal velocity when its driving and dissipation forces (respectively $F_d$ and $F_v$) are balanced:

$$8k\gamma_{oa}\alpha R^2 \propto f_s \mu_o \nu ln\left(\frac{R}{L_o}\right) R \tag{S11}$$

which can be rewritten as

$$v \propto \frac{8k\gamma_{oa}}{\mu_o \ln\left(R/L_o\right)}\frac{\alpha R}{f_s}, \tag{S12}$$

giving a scaling law for droplet velocity on shaped-liquid surfaces with wettability gradients. This result is comparable to the equation obtained by Yang et al. (2008) for a patterned solid surface, with notable differences due to the conformal oil layer and the resulting three-phase contact line.

# 7 Comparison between liquid-surface and super-hydrophobic surfaces

**We demonstrate here that a liquid surface allows to create higher wettability gradient than a super-hydrophobic surface for the same gradient of solid fraction.**

The contact angle for a droplet on a liquid-surface can vary from $\approx 180°$ to $108.4°$, while it only varies from $\approx 180°$ to $165°$ on a super-hydrophobic surface (respectively blue and green curves on Fig. 1 of the main manuscript). The higher difference in contact angle ($71.6°$ for the liquid surface and $\approx 15°$ for the super-hydrophobic surface) allows to create higher wettability gradient for a same gradient of solid fraction.

For example, for a surface with a solid fraction increasing linearly from 0 and 1 on a given length $L$, and a droplet of radius $R$, we can express the force driving the droplet on the liquid surface from equation (S1) and equation (S2):

$$F_{ls} = 2kR(\gamma_{oa} + \gamma_{wo})(\cos\theta_{ls}(x = -R) - \cos\theta_{ls}(x = R)) \tag{S13}$$

$$= 2kR(\gamma_{oa} + \gamma_{wo})\frac{2R}{L}(\cos\theta_{ls,max} - \cos\theta_{ls,min}) \tag{S14}$$

and similarly on the superhydrophobic surface:

$$F_{shs} = 2kR\gamma_{wa}(\cos\theta_{shs}(x = -R) - \cos\theta_{shs}(x = R)) \tag{S15}$$

$$= 2kR\gamma_{wa}\frac{2R}{L}(\cos\theta_{shs,max} - \cos\theta_{shs,min}) \tag{S16}$$

We can then express the ratio of the forces created by the wettability gradients between a liquid-surface and a super-hydrophobic surface from equation (S14) and equation (S16):

$$\begin{aligned}\frac{F_{ls}}{F_{shs}} &= \frac{(\gamma_{oa} + \gamma_{wo})(\cos\theta_{ls,max} - \cos\theta_{ls,min})}{\gamma_{wa}(\cos\theta_{shs,max} - \cos\theta_{shs,min})}\\ &= \frac{(0.0198 + 0.038)(\cos 180° - \cos 108.4°)}{0.072(\cos 180° - \cos 175°)}\\ &= 16.1\end{aligned}$$

Hence, for a similar gradient of solid fraction, the liquid surface allows a higher wettability gradient that results in a driving force about 16 times higher.

# 8 Hanging droplet motion

**We present here additional visualisations of hanging droplets moving due to wettability gradients.**
(See also Movie S4). The droplets are generated using a reservoir of water, whose free-surface is excited at its characteristic frequency with a shaker. Droplets in a given range of size are successfully captured thanks to the high normal adhesion of the liquid surface. Droplets are then self-propelled towards the high wettability regions (right here). Samples are carefully leveled prior to each experiments, to ensure that gravity is not playing any role in the droplets motion.

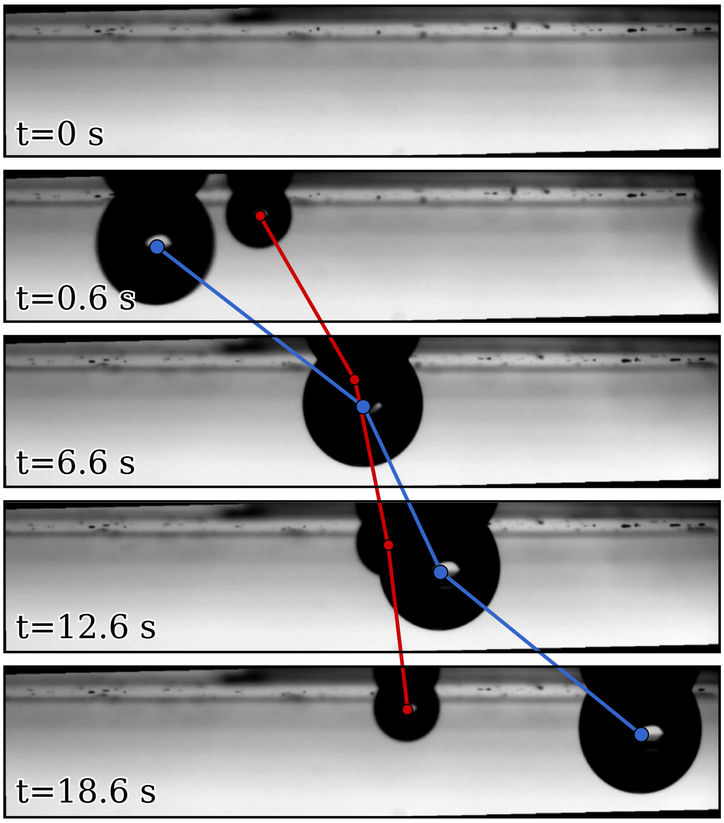


Figure S7: Droplets projected on an upside-down shaped liquid surface. Droplet are captured due to the surface high normal adhesion and driven towards the high wettability region (right here). Droplets of different sizes move at different velocities, in agreement with Fig. 2 from the main manuscript.

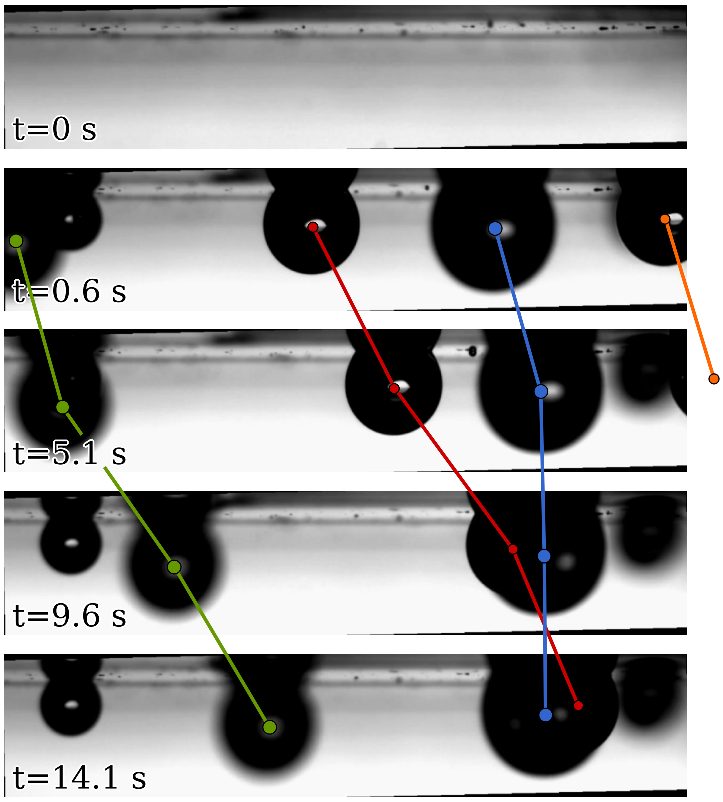


Figure S8: Same as Fig. S7, but for a higher number of droplets. Some droplets lands outside of the shaped region of the sample and remain motionless.

## 9 Transport along a curved path

**We present here a liquid-surface allowing to transport droplets on a curved path.**

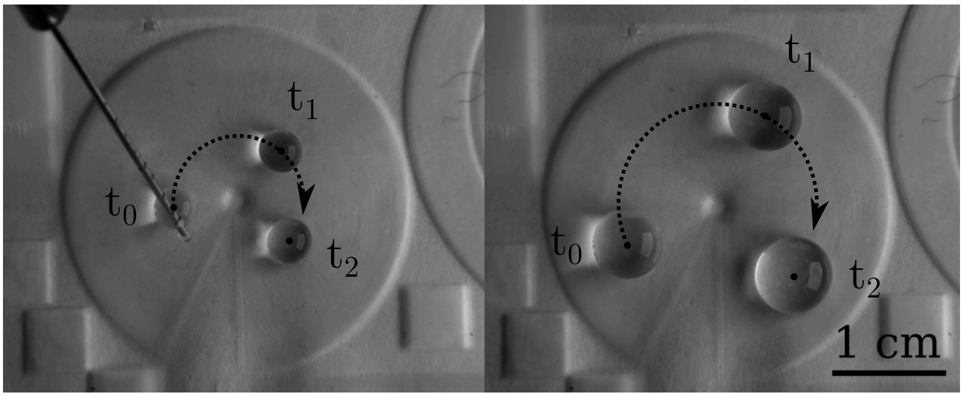


Figure S9: Transport of 5 µl and 10 µl water droplets on a circular wettability gradient. Droplets are deposited on the left at $t_0$ and move along the circular pattern. See also Movie S3.

## 10 Captions of the movies:

**Movie S1.** Droplet rapid motion due to a discontinuity in wettability of a shaped liquid surface. The solid fraction is $f_s = 0.1$ on the left and $f_s = 0.9$ on the right. When released, the droplet moves rapidly towards the region of higher wettability (higher solid fraction). The scale bar is 1 mm.

**Movie S2.** Droplet deposited on a shaped liquid surface with a gradient of solid fraction ($\alpha = 0.05\,\mathrm{mm}^{-1}$). The gradient of wettability and the low pinning force allow the droplet to move towards the high wettability regions. The scale bar is 1 mm.

**Movie S3.** Top view of two droplets deposited on a shaped liquid surface with a gradient of solid fraction along a curved path. Droplets move along the curved rails towards the region of higher wettability. For convenience, movies are accelerated by a factor of 4. The reason why droplets are accelerating in the middle of their trajectories is still to be explained. Our current hypothesis is that the rail curvature have a complex impact on the droplet footprint.

**Movie S4.** Droplets projected on an upside-down shaped liquid surface. A droplet is captured due to the surface high normal adhesion and then driven towards the high wettability region (right here). The camera couldn't be perfectly levelled during measurements and the video have been rotated afterwards to correct this. This explains the presence of the white region at the bottom of the video.
